# Metabolic pathways further increase the complexity of cell size control in budding yeast

**DOI:** 10.15698/mic2014.09.167

**Published:** 2014-08-22

**Authors:** Jorrit M. Enserink

**Affiliations:** 1 Oslo University Hospital, Department of Microbiology, Sognsvannsveien 20, NO-0027 Oslo, Norway.

**Keywords:** metabolism, cell size, cell growth, cell cycle

## Abstract

How organisms regulate their size is a major question in biology. With a few notable exceptions (such as cell divisions in the early embryo), most cells need to reach a critical size in order to initiate a new cell cycle. How cells set a critical cell size, and how they know it has been reached, is not well understood. Using various types of experimental systems, decades ago two main models were proposed for cell size homeostasis: the deterministic model and the probabilistic model.

The deterministic model postulates that cells only enter the cell cycle when they have reached a critical cell size. Early studies clearly showed that most of cell growth occurs in the unbudded (i.e. G1) phase of the cell cycle 1. These studies also observed that upon birth, the daughter cell is smaller than its mother, and that it spends more time in G1 to attain a certain critical size required for cell cycle entry 2. Furthermore, the critical size can be reset by the cell in response to changes in the quality and availability of nutrients 3. In the presence of a relatively poor carbon source, such as glycerol, critical cell size is relatively small. When cells are transferred to a rich carbon source, like glucose, they temporarily arrest in G1 to reset the critical size to a larger size 3. Together, these findings resulted in formulation of a model in which "growth and not the events of the DNA division cycle are rate limiting for cellular proliferation and that the attainment of a critical cell size is a necessary prerequisite for the "Start" event in the DNA-division cycle" 2. In other words, cells have a mechanism for monitoring their size, and only when a critical size has been reached a series of events is initiated leading to cell division 4.

Around the same time an alternative explanation for the relationship between cell size and the cell cycle emerged, referred to as the transition probability hypothesis 5. This probabilistic model divides the cell cycle in two parts, the B phase (consisting of S, G2 and M), and the A phase (consisting of G1). Cells are waiting in the A phase for a random event that triggers transition to the B phase, and they can stay in the A phase for any length of time, but the probability of transitioning into the B phase is constant 6. This model is supported by the observation that cell size at the time of bud emergence (i.e. cell cycle entry) is variable between individual parental cells 7. Later refinements include the sloppy size control (SSC) model, in which the probability of entering the B phase increases with cell size, such that small cells have a low probability and large cells a high probability 7,8. The probabilistic model is supported by observations that the distribution of cell cycle times includes an exponential component that cannot be explained simply by a deterministic model 9,10. However, it was noted that the probabilistic and deterministic models are not necessarily mutually exclusive 11, as demonstrated by studies in *Chlamydomonas* and *S. cerevisiae* 12,13. Thus, both deterministic and probabilistic models can operate in one cell cycle.

One issue that somewhat befuddles the field stems from studies that overly relied on the deterministic model of cell size control, strictly using cell size as a read-out for the timing of Start 14. Here, a gene is classified as an inhibitor of Start when its inactivation results in cell cycle entry with a small cell size. It is inferred that such mutants enter the cell cycle too early (i.e. when the correct cell size has not yet been attained), and that under normal conditions the function of the gene is therefore to suppress Start until the cell has reached its correct size. Conversely, genes that, when mutated, result in cell cycle entry with large cell size are classified as activators of Start. This applies well to cell cycle genes, because mutations in genes that regulate the cell cycle are particularly well correlated with cell size. For instance, deletion of the cyclin *CLN3* delays Start and results in large cell size at the time of cell cycle entry, whereas expression of a hyperactive allele of *CLN3* advances Start, resulting in cell cycle entry at smaller cell size 15,16. This line of reasoning has been successfully applied for identification of novel cell cycle genes simply by systematically screening mutant libraries for deviant cell size. For instance, *whi5*∆ mutants were found to have a small cell size phenotype 14, indicating that under normal conditions *WHI5* functions as an inhibitor of Start. Indeed, *WHI5* was subsequently shown to inhibit cell cycle entry by inhibiting the activity of SBF, a transcription factor complex that is required for cell cycle entry (17,18, for a review see 19). Thus, for *WHI5* there exists an excellent correlation between cell cycle regulation and cell size.

In contrast, other studies have shown that cell size at Start is not necessarily a good proxy for regulation of the timing of Start. For example, deletion of *SFP1*, which encodes a transcription factor for ribosomal protein genes, results in cell cycle entry at very small cell size 14,20. Strictly applying the deterministic model, *SFP1* was therefore classified as a repressor of Start 14. However, when the actual time was measured from birth of the daughter cell to onset of DNA replication (i.e. G1 phase), it turned out that *sfp1*∆ mutants spend an abnormally long time in G1 phase 20. This indicates that *SFP1* is actually required for *promoting* Start, not for repressing it. Independent studies have come to the same conclusion that *SFP1* likely has a positive role at Start 21. Thus, the deterministic-centered idea that cell size at Start alone is a good proxy for G1 progression is too simple. In addition to critical cell size, other factors must also be taken into account, such as cell size at birth, the growth rate during G1 phase, and the potential effect of stochastic events 13,20,21. Further complexity is also added by the fact that in addition to all these intrinsic parameters, environmental conditions also affect cell size, such as the availability and quality of nutrients 3. Despite the fact that nutrients - and thus cell metabolism - play an important role in determining cell size, the involvement of metabolic genes has received surprisingly little attention, possibly because they were deemed less interesting than, say, cell cycle genes. Taken together, the mechanisms that the cell uses to regulate its size are clearly very complex, and we do not yet fully understand all the parameters and players involved in this process.

Against this backdrop, recent studies have begun to characterize some of these important parameters 13,21, focusing in particular on the variability in cell size that commonly occurs at the time of budding, which was first observed decades ago (e.g., 7). Different explanations for this variability have been put forward. For instance, one report showed that cell size variability at Start is in large part due to molecular noise intrinsic to the mechanisms that control the transcriptional program required for cell cycle entry 13. In contrast, others have found that the variation in cell size at Start is not because of an inherent stochastic behavior, but that it is an individual parameter set by the rate at which each individual cell grows during G1 21. The reason for this apparent disagreement is unclear, but it is possible that several parameters have to be analyzed simultaneously in order to get the full picture.

In a recent publication in Microbial Cell 22, Soma *et al*. did exactly that, i.e. measure birth size, the rate of cell growth during G1, the critical cell size and the duration of G1, and all this in the presence of different carbon sources. This approach allowed them to tease apart the effect of metabolism and growth rate on setting of critical cell size. Interestingly, they identified physiological nutritional conditions in which cell size at Start can be set independently of the rate of size increase in G1. They also described several mutants in which critical cell size is uncoupled from the growth rate. For instance, cells lacking *ADK1*, the gene encoding adenylate kinase, have a much lower growth rate during G1 than wild-type cells, yet their critical size is much larger. These findings are at apparent odds with the recently postulated hypothesis that the rate of size increase is a major determinant of critical cell size 21. Soma *et al.* 22 argue that this discrepancy may stem from (i) the fact that an insufficient number of mutants and nutritional conditions were studied by Ferrezuelo *et al.* 21, and (ii) from the use of a different method to calculate increases in cell size 13. Whichever the explanation, while the rate of size increase during G1 may contribute to setting the critical cell size in some cases, the findings by Soma *et al. *clearly demonstrate that this certainly does not hold true for all growth conditions 22.

Another observation by Soma *et al.* is the large phenotypic space occupied by mutations in different metabolic genes, i.e. there did not appear to be a clear correlation between birth size, the rate of size increase and critical size. One explanation is that multiple metabolic pathways control different aspects of these processes. This would not be surprising, given the fact that cell growth and the cell cycle must be tightly regulated to ensure homeostasis. Comprehensive epistasis analysis of larger sets of mutants will be important for determining how these pathways operate to control cell size during G1. Another, less interesting explanation could be that mutations in metabolic genes often have widespread effects on cell physiology, which particularly affect G1-specific events 23. For instance, deletion of the aspartate kinase *HOM3*, which is important for biosynthesis of methionine and threonine, indirectly also affects DNA replication to cause cell cycle defects 24. Such pleiotropy can make it difficult to dissect the exact effects of each pathway on birth size, the rate of cell growth and critical size. Nonetheless, it is clear that metabolic pathways play an important, previously underestimated role in these processes.

In conclusion, how metabolic pathways are wired into the network that sets critical cell size remains mysterious. Unraveling the underlying molecular mechanisms will be a major goal for future work, and the study by Soma *et al.* provides an important framework for these studies.
